# Depression and the nature of Trinidadian family practice: a cross-sectional study

**DOI:** 10.1186/1471-2296-8-25

**Published:** 2007-04-26

**Authors:** Rohan G Maharaj

**Affiliations:** 1 The Unit of Public Health and Primary Care, Department of Para-clinical Sciences, Faculty of Medical Sciences, The University of the West Indies, St Augustine, Trinidad and Tobago

## Abstract

**Background:**

Depression is the most common mental disorder; in an ambulatory-care setting 5 to 10% of patients meet the criteria for major depression. Despite extensive documentation in primary care internationally, Trinidadian studies published on depression have been primarily hospital-based and focussed on suicide. The objectives of this study were to determine the prevalence of depression, the variables associated with depression and the commonest reason for the encounter (RFE) among adult patients attending Trinidadian fee-for-service family practice?

**Methods:**

This was a cross-sectional descriptive survey of consecutive patients taken from a stratified random sample of family practices in the north-west region of Trinidad. To measure depression the Zung scale was modified for use as a brief diagnostic tool. This modified Zung scale, when tested against a psychiatric interview, revealed that at a cut off point of 60, the scale had a specificity of 94% (95% CI 87–100), a sensitivity of 60% (95% CI 45–75), and a likelihood ratio for a positive test result of 10 (95% CI 6–42).

**Results:**

508 patients from 28 practices participated; a response rate of 85%. Participants were primarily younger 18–49 years (66.7%), female (69.5%), and educated, with 72.8% having received a secondary school, technical school or university education. Sixty-five (12.8%) of the respondents (95% CI 9.9–15.7) were determined to be depressed. Chi-square analysis revealed no statistically significant association between depression and age, ethnicity, education levels, occupation or marital status (*p *> 0.05). Binary logistic regression indicated that the likelihood of being depressed (*p *< 0.05) decreased with the increasing age of the patient and was inversely proportional to patient's achieved level of education; and that patients not presently in a relationship were more likely to be depressed than patients who were currently in a relationship. The 508 participants had 630 RFE, with 'check-ups' (17.5%) being the commonest, followed by joint pains (13.4%) and upper respiratory infections (10.5%).

**Conclusion:**

The Trinidadian family physician has to maintain a high index of suspicion in the knowledge that as many as one of every eight adult patients may be depressed and that younger patients of lower educational status who were not currently in a relationship were more likely to be depressed.

## Background

Mental disorders constitute a major problem within populations. A World Health Organisation (WHO) study of mental disorders among 25 000 persons in 14 countries (including Brazil, Chile, China, India, Nigeria, USA and several European nations) revealed that 25% had well-defined disorders, and an additional 9% were sub-threshold. The most common disorders were depression (10%), generalised anxiety disorders (8%) and harmful use of alcohol (3%) [1]. At a population level, as many as one in eight individuals may require treatment for depression in their lifetime [2]. This level of poor mental health carries a large human and economic cost. The World Bank in 1993 (as cited in Craig and Boardman [3]) estimated that mental health problems create 8% of the global burden of disease. This is more than tuberculosis, heart disease or cancer.

With such appalling statistics in population studies it is not surprising to find that mental disease is common in family practice settings, since for many this represents their first contact with the health care system. In Britain, for example, mental disorders comprise about 25% of general practice consultations [3]. Of the mental disorders in primary care, depression is the most common [4]. In an ambulatory care setting, 5 to 10% of patients meet the criteria for major depression [5]. This represents a level of morbidity in primary care that surpasses hypertension, to which much more effort and resources have traditionally been devoted.

Despite this extensive documentation internationally, Trinidadian studies published on depression have been primarily hospital based and focussed on suicide [6-11]. There have also been 3 reports on the adolescent population [12-14], and one each on depression among patients attending chronic disease clinics and patients attending family practice with fatigue as a reason for the encounter (RFE) [15,16]. This present study aims to fill the existing knowledge gap with special consideration of adults attending Trinidadian family practices.

Although, 'most people with mental health problems receive care only from their family physicians' [17], no studies have been done in Trinidad to determine the prevalence of mental illness or depression in family practice. The objective of this study was to determine the prevalence of depression among adult patients attending Trinidadian fee-for-service family practices. Secondary questions include, what are the commonest RFE in Trinidadian family practice? Which demographic features or medical variables are associated with the presence of depression among family practice patients?

## Methods

This was a cross-sectional descriptive survey with consecutive sampling of family practice patients taken from a stratified random sample of family practices in the north-west region of Trinidad. Stratification was done to ensure that all areas within the north-west region of Trinidad would be represented. Although small in size, there are many ethnic and economic nuances within the region chosen. To the west of Port of Spain, the capital city, there is a very affluent population consisting of a complex mix of ethnic groups including many expatriates and business and professional persons. The less wealthy eastern areas within the region are represented by higher percentages of persons of Afro and Indo-Trinidadian descent. Finally in the southernmost part of the region, the area of Chaguanas has a predominantly Indo-Trinidadian population.

There is a multi-tiered health care system in Trinidad and Tobago, with both private and public health care facilities offering primary, secondary and tertiary care services. Private fee-for-service family practice is not exorbitantly priced with most encounters costing between $20–30 (USD). Public clinics, including chronic disease and walk-in clinics at government health offices were excluded, as these clinics either have been studied previously [15] or will be studied in the future.

### Recruitment of physicians

A sample frame of family physicians practising in this region was created from lists available through pharmaceutical companies, the 2003 telephone directory, personal knowledge of practices and lists obtained from medical organisations in Trinidad and Tobago. Stratification of the sample was done according to natural geographical boundaries and a random sample of physicians was taken from this stratified list, so that all populations in this region would be represented. Physicians eligible for inclusion in this study included any private family physician or general practitioner in the north-west region of Trinidad. All selected physicians were sent a letter of invitation to participate, information about the study and copies of the physician and patient's consent forms. This mailing was followed up with a telephone call from the Research Assistant inquiring about the physician's willingness to participate and providing further clarification if required.

### Instrument used

To measure depression we used the Zung scale [18-20]. Though primarily used as a screening tool the Zung scale was modified for use as a brief diagnostic tool. This modified Zung scale was tested against a psychiatrist who was blinded as to the results of the test. The psychiatrist used her clinical assessment based on Diagnostic and Statistical Manual of Psychiatric Illness Version IV-TR criteria. We compared the functioning of the modified Zung scale at different cut-off points, 50, 55 and 60. At 50 and 55 the scale performed inadequately with sensitivities of 70% and 60% respectively and specificities of 79% and 85% respectively. The likelihood ratio for a positive result were 3 and 4 for these cut-offs respectively. At a cut off point of 60, the scale had a specificity of 94% (95% CI 87–100), a sensitivity of 60% (95% CI 45–75), and a likelihood ratio [21] for a positive test result of 10 (95% CI 6–42). This cut-off point was used for this present study [22].

### Recruitment of patients

Consecutive patients attending these family practice clinics were eligible to participate once they fulfilled the inclusion, and did not fit the exclusion criteria. Consenting patients were interviewed regarding their main presenting complaints and the presence of specific symptoms over the previous two weeks as contained in the modified Zung depression scale. Patients were chosen without regard to the number of times they had presented for this particular problem i.e. regardless of being a first or follow-up visit. Excluded from filling the questionnaires were patients attending for food handlers' certificate or driver's permits, young people under the age of eighteen, the very ill (as determined by the research assistant or the doctor), those who refuse and those who previously responded. Those patients attending for insurance physicals or physicals for firearm user permits were also excluded. Patients with a past psychiatric history were not excluded from completing the questionnaire. Simple demographic data were also obtained from the required questionnaire. Variables pertaining to age, gender, ethnicity, educational achievement, occupational status, marital status, and presenting complaints were collected.

### Data collection, analysis plan and ethical issues

Questionnaires were administered and results entered by trained research assistants. This was usually carried out in the waiting room because of limited space at most offices. The interviewers were not personally known by the respondents.

Data was entered into an Excel spreadsheet; all entries were rechecked using the original completed questionnaires. If demographic data was missing that patient's full entry was removed. If entries for the modified Zung scale were missing – no more than 2 entries per patient – the lowest score was applied to those entries. This had to be done for two patients only. Job description/occupations were coded according to criteria from Jamaica [23]. Reasons for the encounter (RFE) were coded according to International Classification for Primary Care [24]. The data was then imported into SPSS (version 12); variables were named and a codebook created. The data was analysed using descriptive statistics to generate frequencies including the prevalence of depression, and chi-square tests were performed to determine if the variables were independent of each other. Multivariate analysis techniques were used to determine which of the following demographic characteristics age, relationship status, ethnicity, employment and highest level of education could be regarded as risk factors for depression among patients in Trinidad who attend family practices. Using these 6 categorical variables (recoded into dichotomous variables) a binary logistic regression model was built to predict the presence or absence of depression in the sample of patients. The model was then used to derive estimates of the odds ratio for each factor to tell, for example, how much likely male patients are to be depressed than female patients.

Ethical considerations in this study revolved around patient autonomy and confidentiality. The information form provided clearly stated the right of the patient to refuse to participate and that their non-participation would in no way affect their medical care. And even if they did agree they may later change their mind. The patient's name was not included on any forms collected for removal from the family physician's offices. Confidentiality was of prime concern during the data collection process and this was stressed to the Research Assistants.

No provision was made for patients with high scores to receive medical attention for their depression. The final summation of scores was often completed away from the family physician's office once the questionnaire was adequately filled in. Data was securely stored in space provided by the Unit of Public Health and Primary Care, The University of the West Indies, St Augustine. Approval for this study was granted by the Ethics committee of the Faculty of Medical Sciences, St. Augustine, Trinidad.

## Results

A total of 28 family practitioners participated and 18 provided information on themselves. Seven were graduates of The University of the West Indies and 15 had been in practice for more than 20 years. Fifteen respondent physicians were male, 14 were over the age of 50, and 9 had received postgraduate education on depression.

599 patients were invited to participate; there were 91 refusals with a response rate of 85%. Participants were primarily younger 18–49 years (66.7%), female (69.5%), and educated, with 72.8% having received a secondary school, technical school or university education. There were roughly equal amounts of Indo-Trinidadian (34.3%), Afro-Trinidadian (29.3%) and persons of mixed descent (32.9%). Of the sample, 51.6% was presently in a relationship-married, common-law or visiting. As expected from the levels of education, 34.9% worked at highly skilled or professional jobs, and 19.3% were skilled or semi-skilled workers. Housewives made up 20.1%.

Sixty-five of the respondents (12.8% (95% CI 9.9–15.7)) were determined to be depressed. Four percent of patients admitted to suicidal ideation almost every day for the last two weeks. Chi-square analysis revealed no associations between depression and age, ethnicity, education levels, occupation or marital status (*p *> 0.05). Further details of participants and chi-square analysis are illustrated in Table 1.

**Table 1:** Presence or absence of depression by demographic and medical variables among adult family practice patients in Trinidad.

**Demographic and Medical variables**	**Not Depressed n (%)**	**Depressed n (%)**	**χ^2 ^(*p *value)**
N 508	443 (87.2)	65 (12.8)	
**Age group (years)**			
18–19	14 (3.1)	4 (6.1)	
20–29	111 (25.1)	20 (30.8)	
30–39	73 (16.5)	14 (21.5)	
40–49	93 (20.9)	10 (15.4)	
50–59	60 (13.5)	8 (12.2)	
60–69	56 (12.6)	5 (7.7)	
70–79	27 (6.1)	4 (6.1)	
80+	9 (2.0)	0 (0)	
**Sex**			1.94 (0.104)
Male	140 (31.6)	15 (23.1)	
Female	303 (68.4)	50 (76.2)	
**Marital status**			
Married	191 (43.1)	20 (30.8)	
Single	151 (34.1)	21 (32.3)	
Separated	9 (2.0)	5 (7.7)	
Divorced	17 (3.8)	2 (3.1)	
Common Law	37 (8.4)	11 (16.9)	
Visiting	2 (0.5)	1 (1.5)	
Widowed	36 (8.1)	5 (7.7)	
**Status of relationship**			0.16 (0.393)
Presently in a relationship	230 (51.9)	32 (49.2)	
Not presently in a relationship	213 (48.1)	33 (50.8)	
**Ethnicity**			3.19 (0.202)
Afro-Trinidadian	136 (30.7)	13 (20.0)	
Indo-Trinidadian	148 (33.4)	26 (40.0)	
All Others	159 (35.9)	26 (40.0)	
**Level of Occupation**			4.76 (0.191)
Professional or highly skilled	160 (36.1)	17 (26.1)	
Skilled or semi-skilled	86 (19.4)	12 (18.5)	
Housewife	83 (18.7)	19 (29.2)	
Unskilled, unemployed or retired	114 (25.7)	17 (26.2)	
**Level of education achieved**			1.15 (0.562)
Less than secondary level	117 (26.4)	21 (32.3)	
Secondary	197 (44.5)	28 (43.1)	
Technical or University level	129 (29.1)	16 (24.6)	
**Number of reasons for the encounter (RFE)**			0.66 (0.260)
One RFE	334 (75.4)	52 (80.0)	
Two or more RFE	109 (24.6)	13 (20.0)	

The results of the binary logistic regression are shown in Table 2. The second and third columns of the table give estimates, and their standard errors respectively, of the regression coefficients. The Wald values (in Column 4) are used to test the null hypothesis: namely, *H*_0 _: *β*_*i *_= 0 as against the alternative hypothesis of 'not zero'. The Wald statistic has a Chi-square distribution with 1-degree of freedom in each case as shown in Column 5. Column 6 gives the odds ratio associated with each of the variables in the model and suggests that age, relationship status and level of education are risk factors for depression among this group but sex, ethnicity and employment status are not. Columns 7 and 8 give lower and upper 95% confidence interval limits for each regression coefficient. With respect to odds ratios for the variables that have been found to be useful predictors of depression, the following can be deduced from the table:

**Table 2:** Results of binary logistic regression to determine the independent variables associated with depression.

	**B**	**S.E.**	**Wald**	**df**	**Odds Ratio**	**95% C.I. for Odds Ratio**	
						Lower	Upper

**Age**							
Less than 50 *vs. *over 50 years	-.275	.095	8.292	1	.760	.630	.916
**Sex**	.354	.323	1.204	1	1.425	.757	2.681
**Relationship Status**							
Presently in a relationship *vs. *no relationship	.150	.073	4.158	1	1.161	1.006	1.341
**Ethnicity**	.113	.079	2.041	1	1.119	.959	1.306
**Employment**	.019	.046	.174	1	1.020	.931	1.117
**Education**							
Low or High education	-.260	.128	4.147	1	.771	.600	.990
Constant	-1.338	1.039	1.659	1	.262		

1. The likelihood of being depressed decreases with the increasing age of patient.

2. Patients not presently in a relationship are more likely to be depressed than patients who were presently in a relationship.

3. The likelihood of depression is inversely proportional to level of education.

Finally the results indicate preference for a reduced model using only the three predictors identified as useful. However this was not attempted with the current data set.

The 508 participants had 630 RFE, with 'check-ups' (17.5%) being the commonest, followed by joint pains (13.4%) and upper respiratory infections (10.5%) next. Hypertensive and diabetic patients represented 4.9% and 3.7% of the population respectively. See Table 3. The 65 cases of depression were distributed between 32 RFE with 11 (16.9%) cases of depression presenting with pains in the joints, 9 (13.8%) with diabetes, 9 (13.8%) for check-ups and 8 (12.3%) with symptoms referred to the upper respiratory tract. Chi-square analysis revealed no statistically significant association between RFE such as wanting a check-up, joint pains, URI symptoms, tiredness, pain, recent loss of a close relative, insomnia, headaches or dizziness and the presence of depression.

**Table 3:** The commonest reasons for the encounter (RFE) in Trinidadian family practice.

**Rank**	**Reason for the encounter**	**Number (%)**
1.	Check up	110 (17.5)
2.	Joint pains	85 (13.4)
3.	Upper respiratory infection	66 (10.5)
4.	Hypertension	31 (4.9)
5.	Administrative	30 (4.8)
6.	Abdominal pain	27 (4.3)
7.	Skin rash	27 (4.3)
8.	Diabetes mellitus	23 (3.7)
9.	Headaches	20 (3.2)
10.	Weakness	18 (2.9)
11.	Pregnancy related	16 (2.5)
12.	Generalized pain	15 (2.4)
13.	Female reproductive system	13 (2.1)
14.	Psychological issues	13 (2.1)
15.	Cough	11 (1.7)
16.	Symptoms related to the urinary system	11 (1.7)
17.	Vertigo/Dizzy spells	10 (1.6)
18.	Eyes	10 (1.6)
19.	Fever	8 (1.3)
20.	Swelling on skin	7 (1.1)
21.	Ears	7 (1.1)
22.	Nose	6 (1.0)
23.	Allergies	5 (0.8)
24.	All others including, 'no reason given'	61 (9.7)

	**TOTAL**	**630**

When the individual responses on the Zung scale for those diagnosed with depression were further analyzed by items pertaining to pervasive affective disorders, 96.9% had experienced sadness 'some, a good part or most or all of the time' over the last 2 weeks. And 70.7% were tearful a 'some, a good part, or most or all of the time'.

When items on the scale dealing with psychological disturbances were studied 87.7%, 81.5% and 75.4% respectively responded 'some, none or a little of the time' when asked if they found themselves enjoying things they used to, if they found their life satisfying and complete or if they were positive about the future.

When items pertaining to physiologic symptoms in the past 2 weeks were examined, 60% had trouble sleeping, 27.7% had weight loss, 26.1% had palpitations, and 13.8% had constipation, a 'good part, most or all of the time'. Seventy seven percent had problems with their appetite and 69.2% had a change in their libido.

When items pertaining to psychomotor retardation were examined 69.2% found it difficult to do things they were accustomed to do and 56.9% were 'restless and can't keep still'.

## Discussion

This paper used a stratified random sample of offices in the North West Region of Trinidad and showed that the prevalence of depression among consecutive patients attending these offices was 12.8%. Also, this paper determined the commonest reasons for the encounter (RFE) in Trinidadian family practices. Chi-square analysis revealed no statistically significant associations between the presence of depression and either age, gender, ethnicity, level of education achieved, level of occupation, or number of presenting complaints among these family practice patients. Results of binary logistic regression indicated that the independent variables associated with depression were age, with younger persons (aged 39 or less) more likely to be depressed; level of education, with persons achieving only secondary education or below, more likely to be depressed; and marital status.

This paper adds to a growing literature that illustrates the mental health burden facing this population. Among adolescents in Tobago, the sister isle to Trinidad, [12], adolescents in Trinidad [13,14], among attendees at chronic disease clinics in the south-west region [15] and general practice attendees with fatigue [16] the prevalence of depression was 10%, 28%, 28% and 66% respectively.

### The international literature

Depression in primary care populations has been well recognized in developed countries. For example, among over 75 000 patients in the US primary care populations and using a cut-off of 55 on the Zung scale the researchers found the prevalence of significant depressive symptoms to be 20.9% [20]. The lower cut-off point used in that study (55) may account for the differences in prevalence rates found between this study and the present one. Further, it was found that those who perceived their health as poor were more likely to have severe depressive symptoms than patients who perceived their health as excellent. Women, those in older age groups, and those with lower levels of education were more likely to have clinically significant depressive symptoms than men, those in younger age groups, and those with higher levels of education. When classified by marital status within each sex, married men and women were the least likely to have clinically significant depressive symptoms [20]. Similarly in Trinidad among patients attending chronic disease primary care clinics, women, the older patients, those with poorer educational status and those with many presenting complaints were more likely to be depressed [15].

The RFE can be a useful clinical marker for depression, for example, Gerber et al. [25], at a teaching medical centre in the USA, studied 1042 consecutive patients screened for depression. The presenting complaints or RFE that discriminated between depressed and non-depressed patients (p < 0.05 level) were sleep disorders, fatigue, the presence of multiple complaints (3+), non-specific musculoskeletal complaints, back pain, patients with amplified complaints, shortness of breath and patients with vaguely stated complaints. While the international research has found clear predictive factors for depression, in the Trinidadian population this lack of statistical associations between depression and common RFE has importance for family practice. This result implies there are no simple markers, except age and educational level achieved, which the Trinidadian family physician can use as a 'red flag' for depression. The family physician has to maintain a high index of suspicion, evaluating each patient on individual merit.

### Study limitations

The study is limited by several factors, firstly, by the use of the Zung scale, which is not recognized as a diagnostic, but as a screening scale. This scale was adapted for use in this present study as a brief diagnostic tool because of availability, the researcher's previous experience with the scale and patient's easy understanding of the language. The scale's language had been modified to local idiom prior to testing, and by comparing with a psychiatric interviewer using DSM IV-TR criteria, we come close to a diagnostic scale. The Zung, does not identify manic depressive episodes or dysthymia and so these diagnoses are possibly all part of the spectrum of mood disorders that his study may have detected but not distinguished between. Further work is needed to elucidate these. Secondly, there is always uncertainty as to the representativeness of the sample for the population in question. There are differences between the proportions of the ethnic groups in this study versus the general population. The Central Statistical Office in Trinidad reports that the ethnic mix consists of 40.3 per cent of East Indian descent, 39.5 per cent African, 18.4 per cent Mixed, European 0.6 per cent, Chinese and Other 1.2 per cent. Table 1 illustrates that this study has a larger proportion of persons who describe themselves as 'Mixed'. Thirdly, every effort was made to ensure that the lists used to create the sample frame of practices were up to date and comprehensive. However, since there is no compulsory enforced registration of practices it is difficult to be certain of the representativeness of the family practices selected.

### Strengths of this study

Two components of this research build confidence in the results, firstly, the validity of the scale for the high cut-off point used, and secondly, the random sample of offices used through the north-west region of Trinidad. Another mechanism by which we might determine the quality of this work is to measure it against standard criteria for quality when considering prevalence studies. One such set of criteria suggests that 11 of 12 criteria were met [26]. There is no information on non-responders among the patients. In fact this component was not built into the study. It is possible that non-responding patients were depressed and found the questionnaire too intimidating. The research assistants reported informally that 1 patient was so affected. If all the patients responded and no more cases were identified the lowest proportion of depressed patients would be 10.8% (i.e. 65 cases/599 total patients).

### Reasons for the encounter (RFE)

The major reason for the encounter (RFE) was the 'check-up'. In an audit published locally, the premier reasons for the encounter were complaints due to the respiratory system (13.3%), gastrointestinal system (12.5%), obstetric and gynaecological problems (11%). Check-ups were 10^th ^on the list accounting for 3.4% of encounters [27]. Similarly research from Barbados found that among the RFE the 'health examination' accounted for 3.6% of health problems [28]. However, US studies suggest that as many as 14.5% of patients attended for a 'General medical examination' [29]. So there are precedents for the results seen in the present study. It may be that 'check-up' may have been an easy response for patients who were uncomfortable discussing personal or difficult issues with a researcher in a public setting of a doctor's waiting room. What evidence do we have for this supposition that many Trinidadian patients have potentially embarrassing problems? Two recent studies may clarify this issue. In 2002, a cross-sectional study of 346 men in 37 Trinidadian family practices found that 53% had some level of erectile dysfunction [30]. Additionally a survey carried out by medical students, also in 2002, revealed that among females attending Trinidadian family practices, their experience of intimate partner abuse was as high as 27% [31].

### The future

The information from this study can be used in several ways. Aside from "check-ups" and joint pains, depression represents the next most common problem in Trinidadian Family Practice. See Table 3. This has important implications for undergraduate medical education, postgraduate training in Family Practice locally and for the delivery of mental health care to the population. Additionally, the scope for future research appears substantial. Questions for future consideration include, what exactly is the nature of this 'check-up'? What is the prevalence of the other mood disorders, such as dysthymia, adjustment disorders or manic-depression which might be subsumed under the diagnosis of depression as detected by this scale? What is the detection rate by physicians? Does the presence of depression have a negative consequence for patients in family practice and primary care and does treatment improve the outcomes for patients in a Trinidadian setting? This has already been shown to hold true in other populations [32-36].

One study from 1999 suggests that dedicated mental health professionals do a better job in caring for these patients [37]. So, should the job of the primary care physicians be recognition and referral? In a developing world setting such as Trinidad, there may never be sufficient mental health professionals to adequately address this issue and so the role of the primary care physician and primary health care worker becomes crucial.

As we move from detection to interventions for depression a recent systematic review of the literature is instructive. This review suggests that the most successful guideline and educational interventions were accompanied by complex organisational interventions. The most impressive results apparently arise from changes within health care systems and 'those with complex interventions that incorporated physician education, an enhanced role of the nurse (nurse-case management), and a greater integration between primary and secondary care (consultation-liaison)'. Telephone medication counselling delivered by practice nurses or trained counsellors was also effective. Simple guideline implementation and educational strategies were found to be generally ineffective [38]. This review is an important one and should guide future interventions and research in the field of depression, both in Trinidad and worldwide.

## Conclusion

There is a burden of depressive disorders in Trinidadian family practice, with about 1 in 8 patients having a depressive disorder and about 4% of participants admitting to suicidal ideation most of the time in the previous 2 weeks. Further this paper suggests that younger patients of lower educational status who were not currently in a relationship were more likely to be depressed. The common RFEs in Trinidadian family practice include 'check-up', joint pains, upper respiratory infections and hypertension.

## Competing interests

The author(s) declare that they have no competing interests.

## Pre-publication history

The pre-publication history for this paper can be accessed here:


